# Hepatitis B in Moroccan-Dutch: a qualitative study into determinants of screening participation

**DOI:** 10.1093/eurpub/cky003

**Published:** 2018-01-15

**Authors:** Nora Hamdiui, Mart L Stein, Ytje J J van der Veen, Maria E T C van den Muijsenbergh, Jim E van Steenbergen

**Affiliations:** 1 National Coordination Centre for Communicable Disease Control, Centre for Infectious Disease Control, National Institute for Public Health and the Environment, Bilthoven, The Netherlands; 2 Department for Health Evidence, Radboud University Medical Center, Nijmegen, The Netherlands; 3 Christian University of Applied Sciences Ede, Ede, The Netherlands; 4 Program Prevention and Care, Pharos: Dutch Centre of Expertise on Health Disparities, Utrecht, The Netherlands; 5 Department of Primary and Community Care, Radboud University Medical Center, Nijmegen, The Netherlands; 6 Centre for Infectious Diseases, Leiden University Medical Centre, Leiden, The Netherlands

## Abstract

**Background:**

Chronic hepatitis B (HBV) leads to an increased risk for liver cirrhosis and liver cancer. In the Netherlands, chronic HBV prevalence in the general population is 0.20%, but 3.77% in first generation immigrants. Our aim was to identify determinants associated with the intention to participate in HBV testing among first generation Moroccan immigrants, one of the two largest immigrant groups targeted for screening.

**Methods:**

Semi-structured interviews were held with first (*n* = 9) and second generation (*n* = 10) Moroccan-Dutch immigrants, since second generation immigrants frequently act as their parents’ brokers in healthcare.

**Results:**

Most participants had little knowledge about hepatitis B, but had a positive attitude towards screening. Facilitators for screening intention were perceived susceptibility to and severity of disease, positive attitude regarding prevention, wishing to know their hepatitis B status and to prevent potential hepatitis B transmission to others. Additional cultural facilitators included fear (of developing cancer), and existing high health care utilization; a religious facilitator was the responsibility for one’s own health and that of others. Barriers included lack of awareness and knowledge, practical issues, not having symptoms, negative attitude regarding prevention, fear about the test result and low-risk perception. A cultural barrier was shame and stigma, and a religious barrier was fatalism.

**Conclusion:**

We identified important facilitators and barriers, which we found, can be interpreted differently. Specific and accurate information should be provided, accompanied by strategies to address shame and stigma, in which Islamic religious leaders could play a role in bringing information across.

## Introduction

Chronic hepatitis B virus (HBV) infection may progress into severe liver disease, such as liver cirrhosis and liver cancer.^1^ Worldwide, the prevalence of chronic HBV (measured by testing hepatitis B surface antigen (HBsAg) in blood) varies widely with the highest prevalence in countries of the African- and South-East Asian region.^2^ In the Netherlands, 0.2% of the general population is HBsAg positive^3^ and each year an estimated 200 individuals die of sequelae of chronic HBV infection.^4^ Among Dutch first generation immigrants originating from intermediate- or high-endemic countries, the HBsAg prevalence was estimated as high as 3.77%.^3^^,^^5^^,^^6^

With 385 761 individuals (2016), Moroccans represent together with Turks (397 471 individuals) the largest immigrant groups in the Netherlands. Of the total Dutch population, 2.3% is Moroccan and 2.3% is Turkish.^7^ There are 168 336 first generation Moroccan-Dutch of which about half live in urban areas: Amsterdam (21%), Rotterdam (12%), Utrecht (8%) and The Hague (8%).^8^ In Morocco, the prevalence of HBsAg is 1.81%.^9^ Two small studies showed lower chronic HBV prevalences among Moroccan-Dutch (0.4 and 0%).^10^^,^^11^ However, a meta-analysis found similar chronic HBV prevalence rates in migrants compared with the prevalence of chronic HBV in their countries of origin.^12^ This suggests that the prevalence among first generation Moroccan-Dutch may be similar to its prevalence in Morocco.

In November 2016, the Dutch Health Council recommended blood testing of all first generation immigrants originating from countries with intermediate or high HBV endemicity to detect chronically infected individuals and refer them for monitoring or immediate treatment. Identification of infected individuals also allows measures to prevent further HBV transmission. The Council proposed two implementation strategies: (i) individual case finding by general practitioners (GPs), and (ii) screening programmes in cities or regions with large immigrant numbers.^13^ In 1989, the Netherlands introduced antenatal HBV screening for pregnant women to prevent mother-to-child transmission.^14^ This means that up to now, no national HBV screening programme, specifically directed at first generation immigrants, was in place.

Because of the recent recommendation, there is no information (yet) regarding the numbers of HBV screening participation among first generation Moroccan-Dutch. What we do know, is that other health-related screening programmes reported lower attendance rates among Moroccan-Dutch compared with indigenous populations.^15–19^ Visser et al.^19^ reported a participation rate in breast cancer screening (1995–2001) of 37% for Moroccan women, significantly lower than the overall attendance of 76%. In 2007–08, the overall attendance at breast cancer screening increased to 83%, but (again) remained significantly lower for Moroccan women (54%).^18^ During the cervical cancer screening (1998–2001), the overall attendance was 55.7%; again less Moroccan women participated (35.9%).^17^

Therefore, eliminating barriers for participation in HBV screening is demanded and requires identification of determinants of screening behaviour among Moroccan-Dutch.^20^ Earlier qualitative studies focussing on other preventive programmes for Moroccan-Dutch reported lack of awareness and knowledge, organizational issues (e.g. too busy), socio-cultural aspects (e.g. low level of education and fear of social isolation), perceived susceptibility and benefits and barriers (e.g. fear of the test result) as important determinants for participation in preventive programmes.^15^^,^^21–24^

Although extensive research was conducted on determinants to participate in HBV screening among Turkish-Dutch,^25^ no study examined these determinants among Moroccan-Dutch. Prior to implementing screening programmes as proposed by the Dutch Health Council, our aim was to identify determinants associated with HBV screening participation among first generation Moroccan-Dutch.

## Methods

### Sampling

We used purposive snowball sampling, since it may allow us to reach and study migrants (a hard-to-reach population). As a disadvantage, it may result in selection bias, as participants’ social networks are not random. To limit this form of bias, we used various sources to approach possible study participants, namely community organizations (community and day care centres, mosques, interest groups and civil support foundations) located in various cities in the Netherlands (i.e. mainly Amsterdam, Rotterdam, Utrecht and The Hague), and assured maximum variation in our sample by including male and female participants from different places and of different ages with various levels of education and Dutch language proficiency.

Although first generation migrants (FGM) are the targeted group for screening, we also interviewed second generation migrants (SGM), since they frequently act as brokers for their (grand-)parents in contacts with the Dutch healthcare system.^26^ FGM were defined as individuals born in Morocco and having at least one parent born in Morocco. SGM were defined as individuals born in the Netherlands and having at least one parent born in Morocco.^27^ Of all study participants, we did not know their HBV screening status prior to interviewing, as we wanted to gather information, independently of their status. New study participants were recruited until data saturation was reached (i.e. no new information was found during the last interviews).

Prior to each interview, participants were verbally informed about the study and asked to sign an informed consent form. Participants received a small (non-monetary) token of appreciation. The medical ethical committee of the UMC Utrecht approved this study [16-621/C].

### Semi-structured interviews

Semi-structured interviews were held by a native Dutch-Berber speaking female researcher in Dutch, Berber or a combination of both, and lasted ∼1 h.

We developed a topic list consisting of questions about potential determinants of the intention to have a HBV blood test. This list was based on a compilation of the Health Belief Model (HBM), the Theory of Planned Behaviour (TPB), the Betancourt’s Model of Culture and Behaviour and literature on determinants for (non-) participating to other screening programmes.^15^^,^^21–25^ Van der Veen et al.^28^ proposed this compilation as a conceptual model for her study among Turkish-Dutch regarding HBV screening participation (see Supplementary figure S1).

Interviews started with broad questions regarding knowledge and awareness of HBV. If participants were unfamiliar with the topic, concise verbal information about HBV, transmission and testing was given. As sexual contact and drug use are often seen as taboo, we did not actively inform participants about these transmission routes. We informed participants about the main transmission routes among Moroccan migrants, which are perinatal transmission and blood contact between family members.^5^ However, if interviewees mentioned sexual contact and/or drug use by themselves, we also discussed these topics. Detailed background information was made available in Dutch through our project website (www.rivm.nl/Onderwerpen/H/Hepatitis_B/MARAZ_onderzoek_hepatitis_B).

Subsequently, we asked questions concerning potential determinants of health behaviour, including topics such as shame and stigma.

At the end of each interview, socio-demographic data of the participants were recorded. Participants were also asked to score their Dutch language proficiency as 1 (poor), 2 (average) or 3 (excellent).

Interviews were audio recorded. During one interview, the tape recorder broke down at the start, and one interviewee refused to be recorded. In these two cases, notes were taken and summarized at the end of the interview.

### Data analysis

Recordings were transcribed ad verbatim and thematically analysed through coded transcripts by NH using ATLAS.ti version 7.5.6. A random 30% of all participants was double coded by MLS, MvdM and JvS, and findings were subsequently discussed to reach consensus about their meaning. Berber interviews were directly translated into Dutch transcripts.

## Results

### Study participants

We included 9 first (FGM) and 10 second generation Moroccan-Dutch migrants (SGM) (see Supplementary table S1). The majority was female (63%), and all participants were Muslim. FGM had a mean age of 47 years compared with 26 years for SGM. Four FGM (57% of all FGM) reported to have completed a medium level of education or higher. Of the SGM, 70% had a medium or higher level of education. FGM and SGM both reported a median Dutch proficiency score of 3.

### Thematic analysis

We extracted the following themes that may influence the intention to have a HBV blood test: (i) awareness and knowledge, (ii) cultural aspects and religion, (iii) practical issues, (iv) fear about the test result, (v) perceived benefits, (vi) perceived social norm and (vii) perceived susceptibility to and severity of disease.

#### Awareness and knowledge

Most participants (*n* = 14) expressed not to know what HBV is, or having insufficient knowledge about the disease. The few participants, who did know what HBV is, mentioned a general lack of medical knowledge within the Moroccan-Dutch community. FGM who were aware of HBV and did have sufficient knowledge about the disease, often associated HBV with acquired immune deficiency syndrome (AIDS), as both are sexually transmitted diseases (see box 1).

Box 1 Quotes belonging ‘Awareness and knowledge’, ‘Cultural aspects’ and ‘Religion’

**Table:** 

Theme	Corresponding quote(s)
Awareness and knowledge	‘It often is ignorance. You do not know what it is, you have no symptoms, and you do not know where it may lead to’ (R2, FGM, M, 52 years)
	‘It [HBV] should be more known. More information should inform people. That is necessary, because nobody talks about it’ (R4, FGM, F, 47 years)
	‘Medical knowledge is often lacking’ (R12, SGM, M, 20 years)
	‘People are not educated, so it [HBV] will be swept under the carpet. Look: Only people who know a bit more and have knowledge about the human body will think: Yes, it should go the right way, because our health comes first’ (R8, FGM, F, 45 years)
	‘I would not just do a test, just like that. As long as I feel nothing, have nothing, and perceive nothing, then I am not having a test’ (R2, FGM, M, 52 years)
	‘It’s as if you are searching for a disease. If you are going to test all kind of things, you are 100% sure that you are going to find something’ (R4, FGM, F, 47 years)
	‘It is basically just like AIDS and that kind of things. It [AIDS] is of course much larger and much more aggressive, much more in the media, but actually, it is all a bit the same. Our culture has a bit of shame for it [STDs]’ (R17, FGM, F, 46 years)
Cultural aspects	“For Moroccans, all diseases are taboo. You have cancer, it is taboo. You have diabetes, it is taboo. They do not want to express it. They do not want to tell it. It does not matter what type of disease it is. It will not be mentioned’ (R1, FGM, F, 45 years)
	‘Moroccans often like to go to the doctor. I think they would like to have the test. Especially when you tell them, you can develop cancer. What Moroccans really do not want to hear, is cancer. So, if you say cancer, they will experience stress and say: Please test me’ (R6, SGM, M, 21 years).
Religion	‘A Moroccan who lives according to the Islam says ‘Listen, I will take it all for granted. I will not have myself tested. Or I do not want any medications.’ Another would say: ‘Listen, Allah has created people to cure each other, so you should also accept those medications or let yourself be treated by the concerned expert’ (R16, SGM, M, age unknown)

Abbreviations: AIDS, acquired immune deficiency syndrome; HBV, hepatitis B virus; STDs, sexually transmitted diseases.

#### Cultural aspects and religion

##### Cultural aspects

Most participants (*n* = 15) expressed that, in general, diseases are taboo in the Moroccan culture. Some SGM participants (*n* = 3) mentioned high health care utilization of their (grand-)parents (FGM) as facilitator, and one SGM participant mentioned fear of developing cancer as barrier for taking the HBV test (see box 1).

##### Religion

Some participants introduced Islam as a topic. According to two participants, people’s way to practice their religion may limit their testing behaviour. On the contrary, others mentioned that it could also stimulate people to test (see box 1).

#### Shame and stigma

##### Association of hepatitis B with sexuality

We explored this theme with participants who knew that HBV can be transmitted sexually. A few female participants (*n* = 2) mentioned a difference between men and women regarding the sensitivity among sexuality and indicated the association of hepatitis B with sexuality as barrier (see box 2).

Box 2 Quotes belonging to ‘Association of hepatitis B with sexuality and drugs’ and ‘Disclosure of hepatitis B status’

**Table:** 

Theme	Corresponding quote(s)
Association of hepatitis B with sexuality	‘Hepatitis B, you would think ‘okay, how do you get that? It is sexually transmittable. That is one of the reasons, but for Moroccans it is also a reason. You can also have done that [sexual contact]. People think wrong very quickly in the Moroccan culture. People are ashamed very quickly and do not talk about such diseases [STDs]. Especially women have that’ (R5, SGM, F, 23 years)
	“I think it [to have a HBV test] will be more difficult for women than for men. It is just taboo. It will not be mentioned. I think that women will not have such a test so easily unless it will be mandatory or when they will notice that their liver does not work very well at a later stage’ (R8, FGM, F, 45 years)
Association of hepatitis B with drugs	‘Hepatitis B sounds a bit scary. I thought: ‘Oh, needles.’ You immediately think about a certain community. About addicts and stuff’ (R19, SGM, F, 38 years)
	‘We, our children, they never use drugs or alcohol or whatever. They do not use each other’s needles. No’ (R8, FGM, F, 45 years)
Disclosure of hepatitis B status	‘They would rather keep it [HBV status] for themselves. People are afraid for what would happen if the ‘outside world’ or doctor would know. One is also not aware of the privacy policy of different municipal organizations’ (R8, FGM, F, 45 years).

Abbreviations: HBV, hepatitis B virus; STDs, sexually transmitted diseases.

##### Association of hepatitis B with drugs

Although hepatitis B was more often associated with sexuality, two female participants mentioned the association with drugs as barrier (see box 2).

##### Disclosure of HBV status

Some participants (*n* = 3) thought people would be afraid about others’ disapproving opinion if they would get to know their HBV status. If someone would be tested positive for HBV, they would only disclose this to their partner and/or a limited number of family members (see box 2).

#### Practical issues

The majority did not express any practical issues that may limit them to have a HBV test. However, a few participants (*n* = 2) could imagine practical issues that may impede HBV testing, such as an insufficient Dutch language proficiency. Additionally, the majority (*n* = 10) mentioned the costs of the test as a possible obstacle (see box 3).

Box 3 Quotes belonging to ‘Practical issues’ and ‘Fear about the test result’

**Table:** 

Theme	Corresponding quote(s)
Practical issues	‘It is just a blood test. It is not as if they [people born in Morocco] must drive far away and should take a lot of effort. Yes, you will probably go to the hospital or general practice centre once. Blood will be drawn’ (R19, SGM, F, 38 years)
	‘I think they [eligible people to have a HBV test] will find it [HBV test] a hassle. […] To discuss it with the doctor I think. Many people have troubles with that [language barrier]. […] Then they need a third person and should ask someone else. They would not do that. They will find themselves a burden, so they would let it go. I think’ (R3, FGM participant, F, 45 years)
	‘It is not a cure, so you are investing to know if you have something first. You can compare it to the MOT test (in Dutch: APK). If your car is riding fine, you would rather not have the MOT test, because everything is okay. You have to pay the hours that someone is looking at your car. You are losing money to know that your car is riding fine. It is comparable. However, if your exhaust is broken, and you will have the MOT test, you would pay 100 euros, 200 euros, because you know you must fix the exhaust’ (R6, SGM, M, 21 years)
Fear about the test result	‘If people would say to me: ‘Come, let’s have you tested for hepatitis B’, I would become scared. People rather know nothing [by not testing] then to have the chance to have a bad test result’ (R14, SGM, F, 18 years)

Abbreviations: HBV, hepatitis B virus; MOT, ministry of transport.

#### Fear about the test result

Fear about the test result was frequently mentioned as a serious barrier (see box 3).

#### Perceived benefits

Most participants (*n* = 13) had a positive attitude towards the HBV test and expressed that it will potentially benefit their health, or of their (grand-)parents. The saying ‘A stitch in time saves nine’ (meaning: ‘Prevention is better than cure’) was often mentioned. Preventing potential HBV transmission to others was seen as an additional benefit (see box 4).

Box 4 Quotes belonging to ‘Perceived benefits’, ‘Perceived social norm’ and ‘Perceived susceptibility to and severity of disease’

**Table:** 

Theme	Corresponding quote(s)
Perceived benefits	‘I don’t think there are disadvantages. If you do not have the disease, you have nothing to lose. You did the test. If you have the disease, you will be treated. If you know you do not have the disease, it is all right. Then at least you know that you do not have it [HBV]’ (R1, FGM, F, 45 years)
	‘You are doing something good for common humanity. You are preventing something’ (R17, FGM, F, 46 years)
Perceived social norm	‘They will become anxious to be exposed to you. Is the disease contagious or can I come close to you or talk to you?’ (R1, FGM, F, 45 years)
	‘Among us [Moroccans], there is a strong social control. When people say ‘It is good to have it [HBV test], because of this and that’, then people will have it [HBV test]’ (R11, FGM, M, 52 years)
	‘I would discuss it [having a HBV test] with my partner to check what he thinks about it. That is it. I do not care about the rest. I do not care about what others think about it’ (R17, FGM, F, 46 years)
	‘If the GP approaches me, I will take it [HBV test]’ (R4, FGM, F, 47 years)
Perceived susceptibility to and severity of disease	‘How great is the chance that they have it [HBV]? You also think ‘If I feel good, a test is not necessary’. Even if you tell me that, they can have it without knowing and feeling something. So no, I would not recommend having the test’ (R6, SGM, M, 21 years)
	‘The most important argument in favour (of the HBV test) is the health risk, but that is also my most important argument against (the HBV test). How great is the risk?’ (R12, SGM, M, 20 years)
	‘Most people think: I don’t have it [HBV]. People therefore do not have themselves tested, but I do think it is important. Especially for people who are born in Morocco’ (R5, SGM, F, 23 years)

Abbreviations: HBV, hepatitis B virus; GP, general practitioner.

#### Perceived social norm

As for the social norm regarding HBV, many participants talked about others (including family and friends) who may avoid the ones with HBV, because of their fear to become infected as well. This social norm regarding HBV may influence the testing behaviour of FGM negatively, as people also mentioned social pressure or control. Other FGM, and to a lesser extent SGM, acknowledged the existence of social norms, but did not want to interact in this social environment. These female participants would rather consult their partner or GP to decide whether they should take the test (see box 4).

#### Perceived susceptibility to and severity of disease

A low-risk perception seemed to be an important determinant of male SGM participants for not recommending the test to their (grand-)parents. This is in contrast of what female SGM participants expressed. They addressed the flawed risk perception of others while stating that this indifference should be fought (see box 4).

## Discussion

This is the first qualitative study identifying facilitators and barriers for participating in chronic HBV screening among FGM and SGM Moroccan-Dutch. Most participants had little knowledge about HBV, but had a positive attitude towards testing. Facilitators were perceived susceptibility to (in women) and severity of disease, positive attitude regarding prevention, the wish to know their HBV status, and to prevent potential HBV transmission to others. Cultural facilitators included fear of developing cancer and high health care utilization. A religious facilitator was the responsibility for one’s health and that of others.

Barriers included lack of awareness and knowledge, practical issues, not having symptoms, negative attitude regarding prevention, fear about the test result and low-risk perception (in men). An important cultural barrier was shame and stigma as (i) diseases are taboo, (ii) hepatitis B may be associated with sexual contact and drug use and (iii) disclosure of HBV status. An important religious barrier was fatalism, which is an attitude emphasizing the subjugation of all events to fate.

Some factors, such as perceived social norm or support, religion and knowledge, seemed to act as facilitator and barrier, as seen in our proposed mechanisms (see Supplementary figures S2–S4). The label ‘being different’ may act as barrier, while social support may stimulate people to have themselves tested. Religion can act as facilitator if one takes responsibility of one’s own health and prevents HBV transmission in the light of the Islam, but as barrier if people interpret their health as predestined and not as something they can control (i.e. fatalism).

Overall, our findings are in line with previous studies that focused on preventive behaviour of Moroccan-Dutch,^15^^,^^21–24^ e.g. lack of Dutch language proficiency,^15^^,^^21^^,^^23^^,^^24^ costs,^23^ and gender differences with respect to HBV risk perception.^29^ However, our study identified specific facilitators and barriers, such as the association of HBV with sexual contact and drugs.

Despite several differences between Moroccan-Dutch and Turkish-Dutch, such as culture,^30^ Dutch language proficiency^7^ and screening participation,^15–19^ it is relevant to compare these groups because of their comparable migration status, religion and socio-economic status. Regarding HBV specific determinants, Van der Veen et al.^31^ found in Turkish-Dutch (religious) responsibility for one’s health, reputation and social support in being tested for HBV as facilitators. This is similar to our findings in Moroccan-Dutch. Both in Turkish- and Moroccan-Dutch, barriers were found to be the association of HBV screening with sexual contact and fatalism. In contrast, Moroccan-Dutch also mentioned drug use as an undesirable association with HBV.

In comparison to Van der Veen et al.,^31^ we did not only focus on socio-cultural determinants and, therefore, found determinants that are guided by the HBM and the TPB. Turkish-Dutch mentioned perceived low efficacy of Dutch health care services as barrier and perceived obligation when being invited for screening as facilitator,^31^ which were not found in our study. This may be related to the low perceived quality of the Moroccan health care compared with the Dutch, while Turkish-Dutch perceive a higher quality of health care in Turkey compared with the Netherlands. Also, as we identified ‘not having symptoms’ as barrier, Moroccans may feel less obligated to participate in screening compared with Turkish-Dutch.

### Strengths and limitations

An important strength of our study is the inclusion of SGM, who were generally more assertive and outspoken. This led to more information on topics, such as sexual contact, drug use and fatalism. Additionally, since SGM frequently act as brokers for their (grand-)parents in contacts with the Dutch healthcare system,^26^ they represent an important group to consider in programmes oriented at screening first generation Moroccan-Dutch. Second, to ensure reliability, data were double coded by a second researcher. Third, we mainly targeted cities with a high density of first generation Moroccan-Dutch, for which the Dutch Health Council also proposed HBV screening programmes.

However, there were also limitations in this study. First, we did not specifically ask participants whether they were screened for HBV. It is possible that HBV screening status influenced our study participants’ opinions and the discussion at large. Second, participants did not have the opportunity to review their transcripts. This may have led to reduced internal validity. As Berber is only a spoken language, transcripts were translated and written in Dutch, which may not be easily read by most FGM participants. Third, selection bias might have occurred, as most participants were women and highly educated. The interviewer was a female Moroccan-Dutch researcher, which may have discouraged Moroccan-Dutch men to participate due to their religiousness and cultural norms. These factors may additionally have restricted men, but possibly also women, in what they expressed during the interviews. Unfortunately, it was not feasible in our time frame and budget to include a male Moroccan-Dutch researcher. Moreover, although we did not explore gender differences explicitly, our findings suggested several, as male SGM participants expressed low HBV risk perception as barrier, female participants mentioned the association of HBV with sexuality and drugs and female participants expressed a gender difference regarding sensitivity among sexuality. These differences are only suggestive because of the qualitative nature of this study. These findings seem to imply the importance of tailored communication strategies based on gender, but further research on gender differences is needed. Furthermore, our finding of determinants acting as facilitator and barrier is challenging for communication strategies. This dual effect shows the intrinsic limitation of choosing and labelling determinants of human behaviour. Determinants are not existing entities in the real world, but are chosen terms, used as metaphors trying to understand health behaviour. Peters and Crutzen argue not to build theories using determinants, but to establish a ‘pragmatic nihilism’ perspective, for which, it is essential to define theories and to develop guidelines to operationalize such determinants.^32^ For developing communication strategies for screening participation, this study provided sufficient information and guidance. For future studies, it would be interesting to follow the approach of ‘pragmatic nihilism’, and recreate and operationalize determinants. Finally, although this study provided insight into determinants of HBV testing behaviour in Moroccan-Dutch, we recommend confirming these results quantitatively in a large representative sample of this population.

### Implications and future research

As the majority of participants lacked awareness and knowledge (i.e. health literacy^33^) regarding HBV, an educational campaign is a cornerstone for participation and should by all means be introduced in Amsterdam, Rotterdam, Utrecht and The Hague. This campaign can be tailored to the needs of Moroccan-Dutch using the obtained knowledge. Moreover, as flawed risk perceptions are present, clear and visual information on the risk of developing chronic HBV and liver cancer should be provided to aid informed decision-making among Moroccan-Dutch.

## Conclusion

This study identified important facilitators and barriers, which require careful consideration when designing and implementing communication strategies. Specific and accurate knowledge provision is important, but should be accompanied by strategies to address shame and stigma. Islamic religious leaders within the Moroccan-Dutch community should, therefore, be informed about hepatitis B and risk (i) to bring information across, (ii) to decrease elements of shame and stigma, leading to more acceptance of HBV screening.

## Supplementary Material

Supplementary Figure S1

Supplementary Figure S2

Supplementary Figure S3

Supplementary Figure S4

Supplementary Table S1

Supplementary Figure Legends
