# Supplementary figures and images for: Hepatitis B in Moroccan-Dutch: a qualitative study into determinants of screening participation
Source: Eur J Public Health. 2018 Jan 15;28(5):916–22. doi: 10.1093/eurpub/cky003 (PMC6148971; doi:10.1093/eurpub/cky003)

**
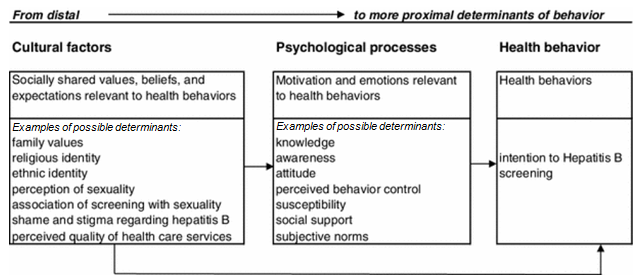
**

Supplement: Supplementary Figure S1 [file cky003_figure_s1.doc]

**
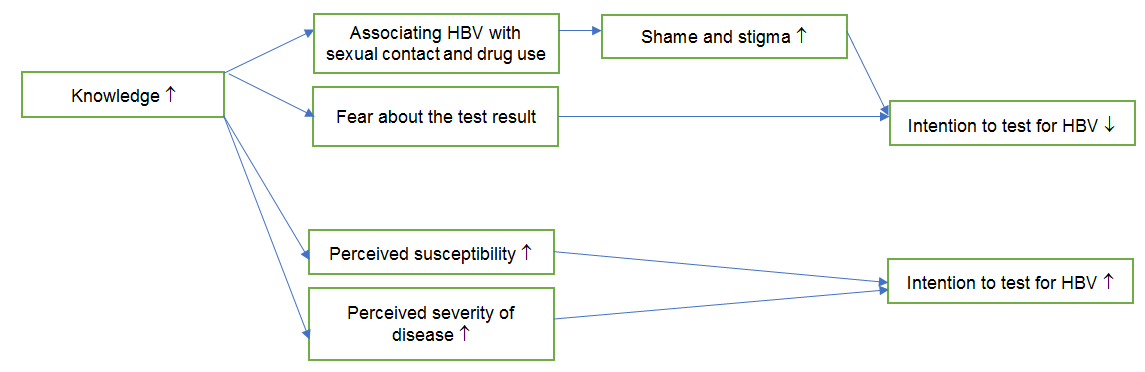
**

Supplement: Supplementary Figure S2 [file cky003_figure_s2.doc]

**
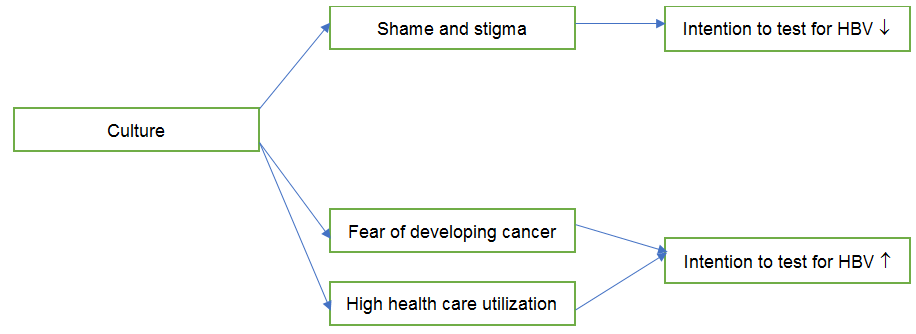
**

Supplement: Supplementary Figure S3 [file cky003_figure_s3.doc]

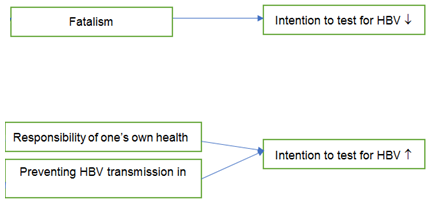

Supplement: Supplementary Figure S4 [file cky003_figure_s4.doc]
