# Supplementary material for: Hepatitis B in Moroccan-Dutch: a qualitative study into determinants of screening participation
Source: Eur J Public Health. 2018 Jan 15;28(5):916–22. doi: 10.1093/eurpub/cky003 (PMC6148971; doi:10.1093/eurpub/cky003)
Supplement: Supplementary Table S1 [file cky003_table_s1.doc]

**Supplementary table**

Table S1. Characteristics of the participants (n = 19)

| Moroccan-Dutch interviewees | Gender (male) | Mean age, in years | Married | Religious | Mean core family size | Highest completed level of education, medium or higher | Main occupation in daily life | | | |
| --- | --- | --- | --- | --- | --- | --- | --- | --- | --- | --- |
|  |  |  |  |  |  |  | Education | Job | Retired | Household |
| FGM  (n = 9) | 3 (33) | 47^a^ | 6 (67) | 9 (100) | 10 | 4 (57)^a^ | - | 3(43)^a^ | - | 4 (57)^a^ |
| SGM  (n = 10) | 4 (40) | 26^a^ | 3 (33) | 10 (100) | 5 | 7 (70) | 6 (60) | 2 (20) | - | 2 (22) |

Data are reported as number of participants (%).

^a^Two participants did not respond to this question.

FGM: first generation migrant, SGM: second generation migrant
