# Supplementary material for: Hepatitis B in Moroccan-Dutch: a qualitative study into determinants of screening participation
Source: Eur J Public Health. 2018 Jan 15;28(5):916–22. doi: 10.1093/eurpub/cky003 (PMC6148971; doi:10.1093/eurpub/cky003)
Supplement: Supplementary Figure Legends [file cky003_figure_legends.doc]

**Figure legends**

Figure S1. Conceptual model of the relationship between social-cognitive, socio-cultural determinants, and hepatitis B-screening intention in Turkish Dutch as proposed by Van der Veen et al. ^(28)^

Figure S2. Proposed mechanisms of the relationship between knowledge and intention to test for HBV

If the level of knowledge would increase and people would know that HBV may be transmitted via sexual contact or contaminated needles, this might lead to shame and stigma within the Moroccan-Dutch population and a decreased intention to test for HBV. More knowledge may also lead to an increased perceived susceptibility and severity of disease, resulting in an increased intention to test for HBV.

Figure S3. Proposed mechanisms of the relationship between cultural determinants and intention to test for HBV

Figure S4. Proposed mechanisms of the relationship between religious determinants and intention to test for HBV
